# Supplementary material for: Bendiocarb and Malathion Resistance in Two Major Malaria Vector Populations in Cameroon Is Associated with High Frequency of the G119S Mutation (Ace-1) and Overexpression of Detoxification Genes
Source: Pathogens. 2022 Jul 23;11(8):824. doi: 10.3390/pathogens11080824 (PMC9330212; doi:10.3390/pathogens11080824)
Supplement: Supplementary file 1 [file pathogens-11-00824-s001.zip › pathogens-1808241-supplementary.pdf]

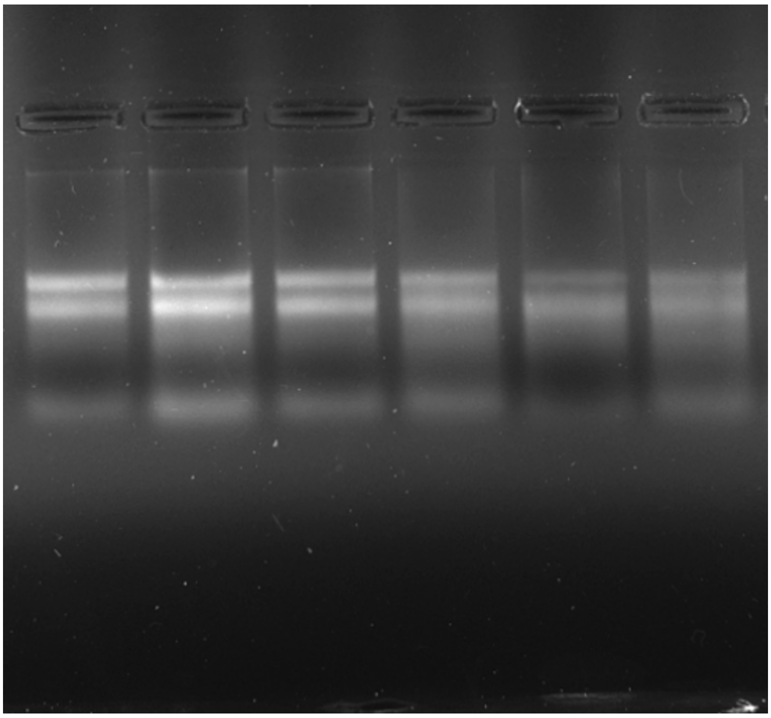

Figure S1. Agarose gel (1.0% w/v) electrophoresis for total RNA of representative samples (N=6). The presence of distinct ribosomal bands (28S, 18S, 5S from top to bottom) and the absence of degradation products shows that total RNA was intact, suitable for downstream analyses. .

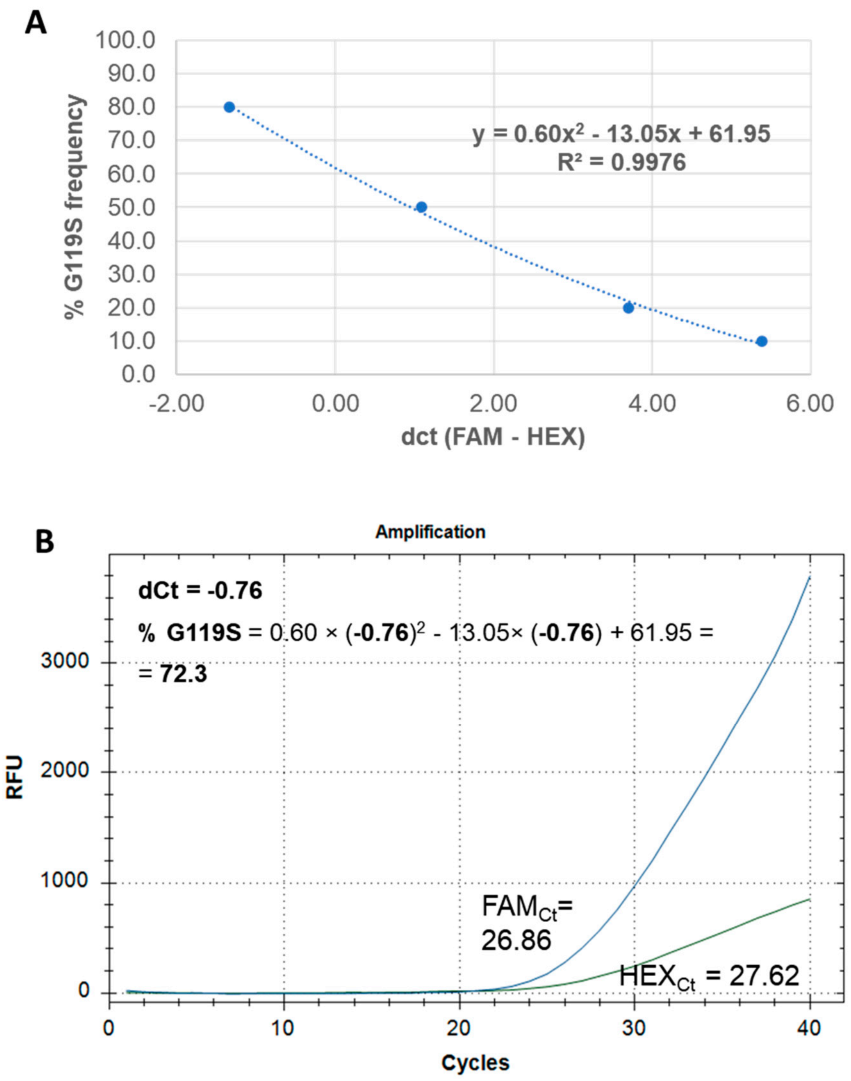

Figure S2. Standard curve for G119S using plasmid sequences with known % frequencies (A) and calculation of % G119S for an unknown population of the study as an example (B).

Table S1 List of primers and probes used for the multiplex resistance gene expression analyses.

**Oligo**	**Assay**	**Sequence (5'-3')**	**Final Concentration (nM)**
*RPS7*_F	Dtx (A)-(D)	CCACCATCGAACACAAAGTTGA	100
*RPS7*_R	Dtx (A)-(D)	TGCTGCAAACTTCGGCTATTC	200
*RPS7*_P	Dtx (A)-(D)	FAM-CCGTGACGTTACGTTCGAATTCCCA-BHQ1	250
*CYP6P3*_F	Dtx (A)	ACAATGTGATTGACGAAACCCT	400
*CYP6P3*_R	Dtx (A)	GGATCACATGCTTTGTGCCG	500
*CYP6P3*_P	Dtx (A)	HEX-ACCCGCGTACCGTCTGTGGACT-BHQ1	350
*CYP6M2*_F	Dtx (A)	CTGGCGTTGAATCCAGAGGT	600
*CYP6M2*_R	Dtx (A)	GATACTTGCGCAGTGATTCATTAAG	400
*CYP6M2*_P	Dtx (A)	ATTO647N-AGAGAAATCCTGCAAAAGCACAACGGAGA-BHQ3	250
*CYP9K1*_F	Dtx (B)	CCGACACGTGGTGATGGATAC	200
*CYP9K1*_R	Dtx (B)	CGTCGTCGGTCCAGTCAAC	400
*CYP9K1*_P	Dtx (B)	HEX-CAATCTTCTGATGCAGGCCCGCAA-BHQ1	300
*CYP6P4*_F	Dtx (B)	CTGGACAACGTTATCAATGAAACC	400
*CYP6P4*_R	Dtx (B)	GCACGGTGTAATCACGCATC	500
*CYP6P4*_P	Dtx (B)	ATTO647N-CCGATCGAGTCACTTTCGCGCG-BHQ3	300
*CYP6Z1*_F	Dtx (C)	CCCGCAACTGTATCGGTCTG	100
*CYP6Z1*_R	Dtx (C)	TTCGGTGCCAGTGTGATTGA	600
*CYP6Z1*_P	Dtx (C)	HEX-TGATGCTGTCCCGATTTAACTTTTCGGC-BHQ1	250
*GSTE2*_F	Dtx (C)	CCGGAATTTGTGAAGCTAAACC	100
*GSTE2*_R	Dtx (C)	GCTTGACGGGGTCTTTCGG	400
*GSTE2*_P	Dtx (C)	ATTO647N-CGGTACGATCATCACCGAGAGCCAC-BHQ3	300
*CYP6P1*_F	Dtx (D)	ACAGGTGGTGAACGAAACCC	100
*CYP6P1*_R	Dtx (D)	GGTGTAATCCTGTCCCGCAA	500
*CYP6P1*_P	Dtx (D)	HEX-CCGCTCGAAACGACGCTGCG-BHQ1	300
*CYP4G16*_F	Dtx (D)	GTCCAAGAAGTTGCGTCGGAC	200
*CYP4G16*_R	Dtx (D)	TCTTCGATTTGCGTTGACGTG	200
*CYP4G16*_P	Dtx (D)	ATTO647N-CTGCAGGCCGACATCATTTTGAAGC-BHQ3	300
